# Pandemic Uncertainty and Socially Responsible Investments

**DOI:** 10.3389/fpubh.2021.661482

**Published:** 2021-03-12

**Authors:** Wenzhong Zhu, Jiajia Yang, Han Lv, Meier Zhuang

**Affiliations:** ^1^School of Business, Guangdong University of Foreign Studies, Guangzhou, China; ^2^Department of Public Policy, King's College London, London, United Kingdom

**Keywords:** pandemics uncertainty, COVID-19 pandemic, COVID-19 uncertainty, socially responsible investments, panel data estimators

## Abstract

This paper examines the effects of pandemic uncertainty on socially responsible investments. We use the overall corporate sustainability performance index in the *Global-100 Most* Sustainable Corporations in the World dataset to measure socially responsible investments. The global pandemic uncertainty is also measured by the World Pandemic Uncertainty Index. We focus on the panel dataset from 2012 to 2020, and the results show that the World Pandemic Uncertainty Index is positively related to socially responsible investments. The main findings remain significant when we utilize various panel estimation techniques.

## Introduction

Socially responsible investment (henceforth SRI) on the stock market means investment strategies that combine social and environmental benefits with financial return. It links many investors' issues, such as social issues, ethical issues, ecological issues, economic issues, etc. Several papers have investigated the difference in financial performance between conventional funds and SRI funds. Some of these studies found no significant difference in the SRI funds' performance among conventional market indices in developed markets, such as those in Australia and Canada (1, 2). However, other results point toward findings that demonstrate significant differences in the SRI portfolios' performance compared to the benchmark market indices. For instance, Brzeszczynski and McIntosh (3) found that the annual average returns of the SRI portfolios (with dividends) were 5.26 and 5.69% higher relative to the benchmarks the Financial Times Stock Exchange (FTSE) 100 and the FTSE4GOOD indices (in their total return versions), respectively. However, the Fama–French–Carhart multifactor models' estimations showed that the SRI portfolio's return needs to consider more factors and cannot always be explained by traditional factors other than market factors. These findings provide new ideas for future research on the SRI stock returns and the SRI portfolios' explanatory factors.

There is another related stream of research that has mainly paid attention to investigating the types of intra-regional transmission and inter-regional transmission of information across stock markets. In the study, researchers have to use stock market indices data to understand the global channels of information transmission across markets worldwide [see, e.g., (4)]. Nevertheless, a small study has been conducted concerning different behavioral finance indicators in the SRI investments. Several research gaps are still needed for promotion. Over time, corporate social responsibility (CSR) investments have become a very important issue, which is of great significance to stock market investors and policymakers because it enhances their understanding of financial markets' interconnectedness (5). In portfolio investment, investors are more inclined to choose social responsibility companies and pay more attention to ethical investment.

We motivate these issues and aim to examine the effects of pandemic-related uncertainties on socially responsible investments. Unlike previous papers, we control pandemic-related uncertainty effects to capture the role of uncertainty on the socially responsible investments nexus.

On the one hand, for factors considered by socially responsible investors for enterprises, Rosen et al. (6) indicated that more individuals and institutions are inclined to invest in companies to support society, which has become a growing trend regarding corporate social responsibility. However, these investors are not willing to sacrifice too much financial return to achieve this even if they value the socially responsible behavior of the companies they invest in Campbell (7). For instance, Kadiyala (8) stated that a typical SRI portfolio shows that the SRI funds can serve as a relatively safe haven during high-risk aversion periods. Still, the evidence supporting this claim is weak in this literature. All in all, the authors show that the continued demand for the SRI funds cannot be attributed solely to purely altruistic motives. Vickman et al. (9) pointed out that uncertainty is considered one-factor affecting investment, and investors need to consider this before making an informed decision. At the same time, contingent and perceived uncertainties need to be taken into account in dealing with finance and other aspects of investment. The combination of macro variables and micro variables creates a more three-dimensional environment in which the valuation methodology provides a framework for the SRI accounting. Berry and Junkus (10) collected many individual investors' independent data and exploited them to infer individual investors' attitudes toward the SRI. It is found that whether investors are inclined to the CSR investments or not, environmental issues are listed as the most important issue.

On the other hand, regarding the CSR investments in the uncertain market environment, Godfrey (11) presented that moral capital can provide shareholders with intangible assets protection similar to insurance, which is based on relationships and helps increase shareholders' wealth. Yanjun and Yong (12) focused on enterprises with negative events as research objects to discuss how to maintain a corporate reputation and how to protect shareholders' wealth in a crisis and expands the research on the role of corporate social responsibility reputation insurance. Take advertising expenditure as an example; Servaes and Tamayo (13) found that enterprises' social responsibility with high customer awareness is positively correlated with enterprise value. Simultaneously, it is pointed out that in the enterprises with poor corporate citizenship reputation, the impact of discovery consciousness on the relationship between the CSR investments and value is the opposite. This view is consistent because corporate social responsibility activities can increase enterprises' value under certain circumstances. Jihui et al. (14) showed that the transfer of funds during market uncertainty has positive economic consequences on corporate social responsibility information and security investment. The fund's safe investment transfer improves the fund's performance and stability and is of great significance for protecting investors' interests. Many studies have shown that the CSR reputation can protect investors' interests and has a certain impact on corporate value.

Additionally, concerning corporate governance during a pandemic, at the beginning of 2020, the new global epidemic had a significant impact on people's way of production and life and a comprehensive impact on people's moral values. It will also have a great impact on the concept and practice of corporate social responsibility. Gefei (15) put forward seven changes brought about by the epidemic to the CSR investments, including the responsibility of employees in basic positions becoming an important focus for enterprises to fulfill their social responsibility in the future, companies paying more attention to reducing negative impacts on the economy, society, and the environment, or avoiding secondary social responsibility problems in solving urgent problems and so on. At this stage, several papers have pointed out changes in the role of business in society during the epidemic. For instance, Zhen et al. (16) said that enterprise is not only important to market entities, promoting the development of high quality in the new period, because of their economic properties and microstructure but can effectively participate in public social management and promote the progressive development of the national systems of governance. Therefore, the enterprise should take social responsibility, and this issue has become part of the COVID-19 era.

There are previous papers that examine the determinants of SRI during the COVID-19 era. For instance, Huo et al. (17) indicated that the spreading threat of COVID-19 could be reduced by researching corporate social responsibility activities and the corresponding measures. Much of the literature has pointed out that social responsibility should be integrated into the corporate governance structure and become the bottom line of enterprise operation during pandemics, including COVID-19. Brammer et al. (18) discussed socially disruptive extreme events, such as the COVID-19, and argued they significantly affect the role of business in the United States. Crane and Matten (19) also indicated that the COVID-19 pandemic has significantly changed the CSR concepts and practices, including its political economy, societal risk, stakeholders, and supply chain responsibility. Garcia-Sanchez and Garcia-Sanchez (20) used the data of the large Spanish companies to determine the objectives of the companies during the COVID-19 pandemic. The authors found that several firms protected the interests of shareholders and investors. Na et al. (21) also discussed the performance of the dilution of corporate social responsibility. It is suggested that enterprises should strengthen the correct values and so on, and guide enterprises to balance profit orientation and social responsibility. He and Harris (22) also discussed several ways in which COVID-19 will change the CSR. Particularly, marketing behavior is significantly affected by the COVID-19 pandemic.

Given this background, we examined the effects of pandemic-related uncertainty on CSR investments. Our main hypothesis is that uncertainty related to the pandemics are positively associated with CSR investments. To the best of our knowledge, ours is the first paper to examine the effects of pandemic-related uncertainty on CSR investments. For this purpose, we used the World Pandemic Uncertainty Index (WPUI) of Ahir et al. (23). We found that the WPUI is positively related to CSR investments.

The remainder of the study is organized as follows. **Data, Model, and Methodology** section defines the data, sets the empirical model, and explains the methodology. **Empirical Results** section discusses the empirical results. **Conclusion** section concludes.

## Data, Model, and Methodology

### Data

This study used the 'Global-100 Most Sustainable Corporations in the World' dataset, which is available at (www.global100.org). We selected the world's top 100 SRI companies from the list to analyze the relationship between the “overall score” and the pandemic uncertainty from 2012 to 2020. The selection of the period is related to the data availability. The overall score is based on the annual ranking of corporate sustainability performance (CSP). The ranking is based on publicly disclosed data (e.g., financial filings and sustainability reports). The “overall score” commutation is based on 17 key performance indicators (KPIs), such as clean revenue, covering resource, employee, financial management, and supplier performance. The detailed methodology can be accessed from https://www.corporateknights.com/reports/2021-global-100/2021-global-100-ranking-16115328/.

The list of the 'Global-100 Most Sustainable Corporations in the World' is a new and unique data set that has never been used in previous research. It was published every January before the World Economic Forum (WEF) at Davos. This list was initiated by Corporate Knights Inc. We used this list because it classifies international CSR companies. These companies make a list because they have demonstrated better ability and better corporate ethics than their peers to identify and effectively manage factors such as physical environment and social governance. Overall, we use the overall index of the CSP as the dependent variable in the panel data estimations. We focused on the country of the corporations.

Moreover, the “World Pandemic Uncertainty Index” is obtained from Ahir et al. (23). The dataset is downloaded from the website https://worlduncertaintyindex.com/data/. We use the aggregate World Pandemic Uncertainty Index (WPUI). The original data are provided for 143 countries from 1996 to 2020. The original dataset is defined at quarterly frequencies. We use the annual frequency data, which is the sum of the quarterly WPUI values. The WPUI index is created by counting the number of times uncertainty is mentioned within proximity to a word related to pandemics in the Economist's Economist Intelligence Unit (EIU) country reports. At this point, the WPUI is the percentage of the word that is “uncertain,” and the variant words related to uncertainty are related to the pandemic terms in the EIU country reports. The ratio has been multiplied by 1,000. It is important to note that a greater value of the WPUI indicates a higher level of uncertainty related to pandemics and vice versa (23).

### Model and Estimation Methodology

The relationship between the Corporate Sustainability Performance (CSP) index and the World Pandemic Uncertainty Index (WPUI) is based on the fixed-effect estimations. The baseline model can be specified as follows:

(1) $$\begin{array}{r} CSP_{i,t}=\alpha_{i}+\beta WPUI_{i,t}{+\theta_{t}+\varepsilon }_{j,t}\;\left(1\right) \end{array}$$

where α_*j*_ is the country-fixed effects, and θ_*t*_ is the time fixed-effects. ε_*j,t*_ is a random error term. We estimate models with robust standard errors, which are clustered at the country level as errors may be correlated within countries. Along with the fixed-effects estimations, we also consider the Ordinary Least Squares (OLS) and the random-effects estimations. Table 1 shows the summary statistics.

**Table 1:** Description of summary statistics.

**Variables**	**Observation**	**Mean**	**Std. Dev**.	**Min**.	**Max**.
World Pandemic Uncertainty Index (WPUI)	693	0.0638	0.0993	0.0000	0.3894
Corporate Social Responsibility (CSR) Index (Overall Score)	693	0.5922	0.0931	0.1042	0.8519

The cross-sectional dependence may be a problem, so the null hypothesis in the Lagrange Multiplier (LM) test of independence is that residuals across entities are not correlated and tested. The *p*-value is < 0.05, so we need a model with cross-sectional dependence.

However, if we found evidence of the cross-sectional dependence in the panel dataset, we need to apply heterogeneous panel estimators as follows.

At this stage, we estimate the following simple model: for *i* = 1, …, *N* and *t* = 1, …, *T* let

(2) $$\begin{array}{r} CSP_{it}=\beta_{i}WPUI_{it}+u_{it} \end{array}$$

(3) $$\begin{array}{r} where\;u_{it}=\alpha_{1i}+\lambda_{i}f_{t}+\varepsilon_{it} \end{array}$$

(4) $$\begin{array}{r} WPUI_{it}=\alpha_{2i}+\lambda_{i}f_{t}+y_{i}g_{t}+e_{it} \end{array}$$

where *WPI* and *TS*_*it*_ are observable variables, β_*i*_ is a country-specific slope of an observable regressor, and *u*_*it*_ captures the unobservable variables. The error term is denoted by ε_*it*_. Note that ε_*it*_ and *e*_*it*_ are assumed to be a white noise process. The unobservable variables in Eq. 3 are defined by the country fixed-effects and α_1*i*_, captures the time-invariant heterogeneity across the countries and an unobserved common factor *f*_*t*_ with the heterogeneous factor loadings. At this stage, λ_*i*_ captures the time-variant heterogeneity across the countries, and it models possible cross-section dependence.

It is important to note that the factors *f*_*t*_ and *g*_*t*_ are not only based on the linear approach over the period under concern. These factors can also be non-linear and non-stationary, with certain implications for possible cointegration analysis. At this stage, we implement various unit root tests with structural breaks. We observe the variables' stationarity. However, some biases can occur because the regressors are driven by several common factors in the observable variables. That is, the presence of *f*_*t*_ in Eqs 3, 4 provides potential endogeneity in the estimations as was indicated by Coakley et al. (24) and Eberhardt and Teal (25). We also use the Common Correlated Effect Mean Group (CCEMG) estimator of Pesaran to solve this potential endogeneity problem (26).

## Empirical Results

Table 2 reports results for the OLS (Column 1), the fixed-effects (Column 2), and the random-effects (Column 3). The OLS result shows that there is a positive relationship between CSR and the WPUI. At this stage, these results are the same with either the fixed-effects and the random effects. The *p*-value for the Hausman test is 0.0523; therefore, the fixed-effects method was used as the benchmark model in the analysis. To see if the time fixed-effects are needed, we performed a joint test to see if the dummies for all years are equal to 0. If they are, then no time fixed-effects are needed. We rejected the null that the coefficients for all years are jointly equal to zero (as *p*-value is 0); therefore, the time fixed-effects are needed in this case (see Table 2, Column 4). The coefficient for the WPUI is 1.078, indicating that the WPUI increase by 1 unit leads to a 1.078 unit increase in the CSR (see Table 2, Column 4).

**Table 2:** Results of the fixed-effects and the random-effects estimations.

	**(1)**	**(2)**	**(3)**	**(4)**
**Variables**	**OLS**	**Fixed-Effects**	**Random-Effects**	**Fixed-Effects**
WPUI	0.126^***^	0.137^***^	0.133^***^	1.078^***^
	(0.0251)	(0.0238)	(0.0237)	(0.179)
2012				−0.0763^***^
				(0.0159)
2013				−0.000701
				(0.0158)
2014				−0.173^***^
				(0.0155)
2015				−0.0246^**^
				(0.0115)
2016				−0.157^***^
				(0.0198)
2017				−0.378^***^
				(0.0611)
2018				−0.0595^***^
				(0.0140)
2019				−0.0791^***^
				(0.0115)
2020				−0.0843^***^
				(0.2314)
Constant Term	0.578^***^	0.577^***^	0.578^***^	0.572^***^
	(0.00546)	(0.00387)	(0.00699)	(0.00995)
Observations	693	693	693	693
R-squared	0.027	0.053		0.483
Number of Panel Member		100	100	100

*The dependent variable is the CSP index to measure the CSR. The robust standard errors in parentheses*.

*** p < 0.01 and

** *p < 0.05*.

Because the cross-sectional dependence was found therefore we perform a panel test with cross-sectional dependence, and the results are presented in Table 3. We also performed the slope homogeneity test in panels following Pesaran and Yamagata (27). The null hypothesis of the test is homogenous slopes, implying that all slope coefficients are identical across cross-sectional firms. However, the test statistics showed a value of −5.79, implying that all slope coefficients are not identical across the cross-sectional firms.

**Table 3:** Results of the heterogeneous panel estimations.

	**(1)**	**(2)**	**(3)**
**Variables**	**MGE with Time Trend**	**MGE with Weighted Average**	**CCEMG Estimator**
WPUI	0.220^***^	0.123^***^	0.119^**^
	(0.0555)	(0.00528)	(0.0526)
Time Trend	0.0127^***^	0.0137^***^	
	(0.00109)	(0.000464)	
Constant Term	0.440^***^	0.445^***^	0.0299
	(0.0121)	(0.0102)	(0.0475)
Observations	680	680	689
Number of Panel Member	95	95	98

*The dependent variable is the CSP index to measure the CSR. The robust standard errors in parentheses*.

*** p < 0.01 and

** *p < 0.05*.

However, the OLS, the fixed-effects, and the random-effects estimations can be biased due to the heterogeneous slope coefficients. As the robustness is checked, we implement several panel time-series estimators that allow for heterogeneous slope coefficients across group members and correlate with panel members. Column 1 in Table 3 reports the Pesaran and Smith's (28) Mean Group Estimator (MGE) with each group-specific regression to be augmented with a linear trend term. Column 2 in Table 3 constructs the coefficient weighted averages across panel members following the weighting method in Verardi and Croux (29). Finally, Column 3 in Table 3 implements the Pesaran (26) CCEMG estimator (26). The result indicates that the WUPI increase by 1 unit leads to a 0.119-unit increase in TS (see Table 3, Column 3).

## Conclusion

This paper examines the effects of the pandemic uncertainty on socially responsible investments. We focused on the world's top 100 SRI companies from the list to model the “overall score” from 2012 to 2020. The overall score is based on the annual ranking of corporate sustainability performance (CSP). The pandemic uncertainty is also measured by the WPUI of Ahir et al. (23). We observe that the WPUI is positively associated with the overall index of the CSP investments. We utilize different econometric techniques, such as the OLS, the fixed-effects, the random-effects, the MGE with the time trend and the weighted average, and the CCEMG estimations. The main finding is robust and used different estimation techniques. This finding is in line with the results of Garcia-Sanchez and Garcia-Sanchez (20). Our findings show that CSP investments are positively associated with extreme events, such as pandemics.

Our findings show that top-100 firms have provided a significant commitment to society and have implemented social responsibilities to reduce pandemics' outcome. However, it is important to note that our findings are limited from 2012 to 2020, and our data capture the effects of the COVID-19 on social responsibility investments. Future studies can include the post-COVID era data and re-examine the global pandemics' effects on corporate responsibility indicators.

## Data Availability Statement

Publicly available datasets were analyzed in this study. This data can be found here: https://www.corporateknights.com/reports/2021-global-100/2021-global-100-ranking-16115328/; https://worlduncertaintyindex.com/data/.

## Author Contributions

WZ: writing original manuscript and project administration. JY: writing original manuscript and estimations. HL: data collection and rephrasing the manuscript. MZ: conceptualization and writing original manuscript. All authors contributed to the article and approved the submitted version.

## Conflict of Interest

The authors declare that the research was conducted in the absence of any commercial or financial relationships that could be construed as a potential conflict of interest.
